# Effect of Food Emulsifiers on Aroma Release

**DOI:** 10.3390/molecules21040511

**Published:** 2016-04-22

**Authors:** Jia-Jia Li, Man Dong, Yan-Long Liu, Lu-Lu Zhang, Yan Zhang, Zi-Yu Yang, Jing-Nan Ren, Si-Yi Pan, Gang Fan

**Affiliations:** Key Laboratory of Environment Correlative Dietology, Ministry of Education, College of Food Science and Technology, Huazhong Agricultural University, Wuhan 430070, China; lijiajia@webmail.hzau.edu.cn (J.-J.L.); dongman@webmail.hzau.edu.cn (M.D.); ilsdln@163.com (Y.-L.L.); zhanglulu@webmail.hzau.edu.cn (L.-L.Z.); zhangyan1105@webmail.hzau.edu.cn (Y.Z.); yangziyu@webmail.hzau.edu.cn (Z.-Y.Y.); renjingnan@webmail.hzau.edu.cn (J.-N.R.); pansiyi@mail.hzau.edu.cn (S.-Y.P.)

**Keywords:** emulsifiers, xanthan gum, aroma release, SPME, GC-MS

## Abstract

This study aimed to determine the influence of different emulsifiers or xanthan-emulsifier systems on the release of aroma compounds. Solid-phase microextraction (SPME) and GC-MS were used to study the effects of varying concentrations of xanthan gum, sucrose fatty acid ester, Tween 80 and soybean lecithin on the release of seven aroma compounds. The effects of the emulsifier systems supplemented with xanthan gum on aroma release were also studied in the same way. The results showed varying degrees of influence of sucrose fatty acid ester, soybean lecithin, Tween 80 and xanthan gum on the release of aroma compounds. Compared with other aroma compounds, ethyl acetate was more likely to be conserved in the solution system, while the amount of limonene released was the highest among these seven aroma compounds. In conclusion, different emulsifiers and complexes showed different surface properties that tend to interact with different aroma molecules. The present studies showed that the composition and structure of emulsifiers and specific interactions between emulsifiers and aroma molecules have significant effects on aroma release.

## 1. Introduction

Flavour plays a very important role in the preference for and consumption of foods [1]. Among sensory evaluation components such as colour, rheological properties, or texture, flavour plays an important role during eating [2]. Allowing a controlled release of aroma from food systems has often been a top priority for manufacturers. Food flavourings are important food additives that have become essential in almost all processed foods. To improve the aroma and the unique flavour of food, natural or synthetic flavours are often added during food production. However, a lot of the aroma compounds in food is lost during transport and storage, which affects the quality of food [3,4]. Emulsifiers, thickeners, other additives in food systems, and even food itself are emulsions. In order to preserve the maximum amount of aroma, the effect of emulsifiers on aroma release was studied.

Many studies have been performed to understand the theory of aroma release from food, which delays the release of aroma compounds and enhances the flavour of food [5,6]. The binding interactions among aroma compounds, emulsifiers and partition ratios among different phases are of great importance on the aroma release of foods. This determines the correlative release of flavour compounds during processing, storage and consumption [7,8,9]. The changes of the concentration of the released aromas result from the binding between flavours and polymer molecules and from the system viscosity. The reduction of flavour release is caused by hydrogen bonding, formation of inclusion compounds, or hydrophobic interactions, according to previous studies [10].

A food emulsifier may be defined as a heterogeneous system of two non-mixing liquid phases, where one of the liquids is dispersed into the other phase [11]. As the surface contact between oil and water is unfavourable, kinetically stable emulsions can be produced by the addition of emulsifiers [12]. Emulsifiers can be used to overcome the activation energy of a system by reducing the interfacial tension between the two layers, and thus enhancing its stability over a long time [13]. Food emulsifiers are also a type of surfactant that can effectively reduce the interfacial tension of the system; these emulsions can stabilize two phase or multiphase systems, including liquids, gases, and particles, which are generally immiscible. The most important functions of food emulsifiers include emulsification, stability, dispersibility, and foaming. Emulsifiers control the retention or release of aroma during food processing and storage, which thereby ensures the flavour and taste of food. Adding different emulsifiers to different aroma compounds can effectively improve product quality.

Emulsifiers are categorised into natural and synthetic emulsifiers based on their HLB values (hydrophilic and lipophilic) [14], which in turn depend on the molecular composition of the emulsifier. The HLB value is also called the hydrophile-lipophile balance value, indicating the equilibrium relationship between the hydrophilic groups and the lipophilic groups in the surfactant molecules. The greater the HLB value is, the stronger the hydrophilicity is, the lower the HLB value is, the stronger the lipophilicity is. Emulsifiers show strong hydrophilicity when the HLB value is high, and a large quantity of hydrophilic groups (such as –OH functions) is present in emulsifier molecules. The following paragraphs give examples of relevant food emulsifiers.

Soybean lecithin is a byproduct from the production and processing of soybean oil. Specifically, it is viscous crude phospholipid with a high oil content and is mainly used as an emulsifier, moisturizer, and thickener in industrial applications. Soybean lecithin is a natural emulsifier that is not only used as a food additive with external activity, but also has important physiological functions. Phospholipids have both hydrophilic and hydrophobic properties because of their special structure. Alkyl radicals of fatty acids make up the hydrophobic part, whereas phosphoric acid and choline constitute its hydrophilic part [15]. 

Sucrose fatty acid esters are the products of the esterification reactions between sucrose and fatty acids. Their emulsifying properties are excellent, with a wide range of HLB values, and they are easily soluble in ethanol and acetone. High monoester content results in strong hydrophilicity, whereas high diester and tri-ester content results in strong hydrophobicity. Sucrose fatty acid esters have a wide range of HLB values (2–16) because of the different degrees of substitution of the sucrose hydroxyls; thus, a series of sucrose fatty acid ester products are obtained. Lipase cannot hydrolyze sucrose fatty acid esters when the degree of substitution exceeds 6; and because its taste is almost the same as that of fat [16]. Sucrose fatty acid esters can be used as a food fat substitute to develop low-fat foods suitable for obese people. 

Tween 80 is a nonionic surfactant and a widely used synthetic emulsifier. Tween 80 is usually utilized in the preparation of oil-in-water emulsions or creams in medicine. It can be used as a co-emulsifier for intravenous injections, and it can dissolve some volatile oil materials as the co-solvent. Tween surfactants have hemolytic effects, and Tween 80 is the weakest among them; at the same time, Tween 80 also poses allergenic problems [17]. The synthesis of Tween 80 includes the dehydration of sorbitol, esterification of oleic acid, and addition of epoxyethane. This suggests that Tween 80 has a very complex composition.

Xanthan gum is a thickener and a microbial polysaccharide with a main chain backbone made of β-(1,4)-d-glucopyranosyl units and side chains with two mannose and one glucuronic acid between two mannose units. In aqueous solutions, xanthan gum experiences a conformational change from an ordered double helix to a random coil through the action of temperature and ionic strength. Xanthan gum can dissolve easily in both cold and hot water, has good viscosity and suspension properties, and is stable in acid or alkaline systems; thus, xanthan gum is often utilized as a thickener, stabiliser, and emulsifier during food processing [18,19,20].

Although the release of single aroma compounds has been widely studied, the effects of emulsifiers, or their combination, with xanthan gum on the release of various aroma compounds are poorly understood. Therefore, this research project was carried out. The main contributions of this study are as follows: (1) xanthan gum, Tween 80, sucrose fatty acid ester, and soybean lecithin were respectively prepared as different solutions with concentrations of 0.1%, 0.2%, 0.3%, 0.4%, and 0.5%. Defined amounts of aroma materials were added, and after a certain processing procedure, the relevant data were obtained by GC-MS analysis; (2) sucrose fatty acid ester, soybean lecithin, and Tween 80 were confected into 0.3% solutions, to which 0.1%, 0.2%, 0.3%, 0.4%, and 0.5% xanthan gum were added. Then aroma materials were separately added and the corresponding data were obtained by GC-MS analysis; (3) finally, the experimental data was collected and processed to analyse it and draw our conclusions.

## 2. Results and Discussion

### 2.1. Influence of Single Emulsifier System on Aroma Release

**Figure 1:**
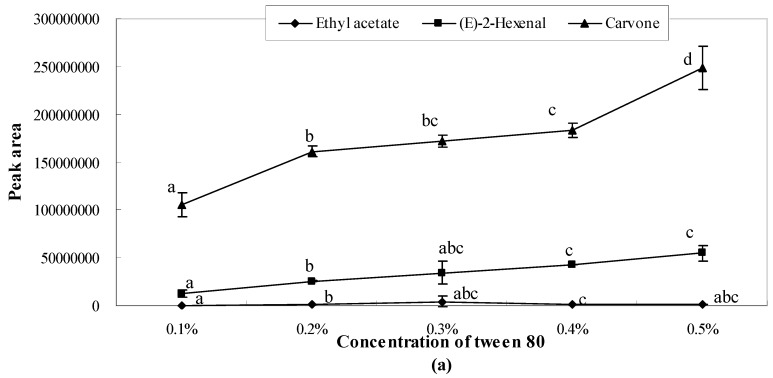

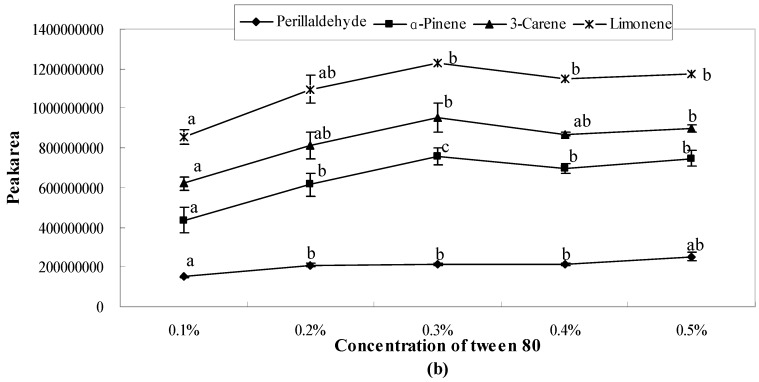
Effects of Tween 80 on the release of ethyl acetate, (*E*)-2-hexenal, carvone (**a**) and perillaldehyde, α-pinene, 3-carene, limonene (**b**). Significant differences (*p* < 0.05) between different concentrations are performed by letters on the same polylines. Where the letters on the same polyline are the same, there is no significant difference between different concentrations.

**Figure 2:**
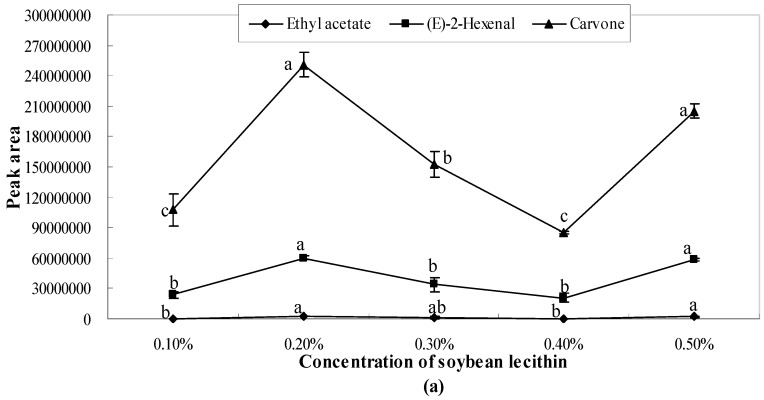

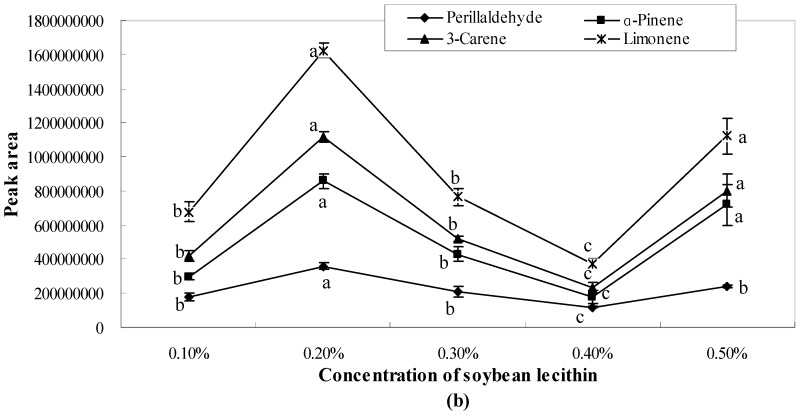
Effects of soybean lecithin on the release of ethyl acetate, (*E*)-2-hexenal, carvone (**a**) and perillaldehyde, α-pinene, 3-carene, limonene (**b**). Significant differences (*p* < 0.05) between different concentrations are performed by letters on the same polylines. Where the letters on the same polyline are the same, there is no significant difference between different concentrations.

**Figure 3:**
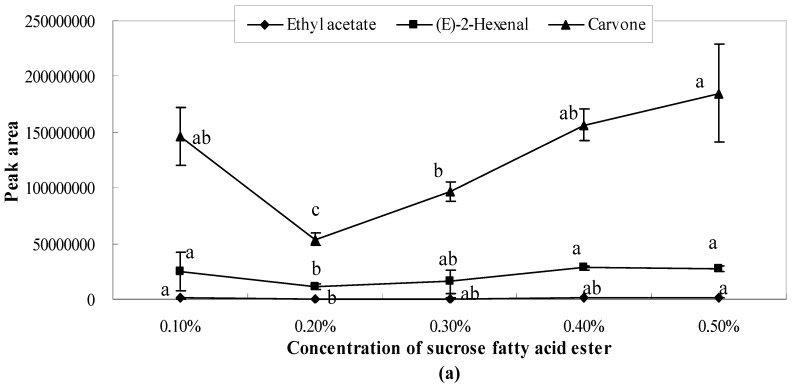

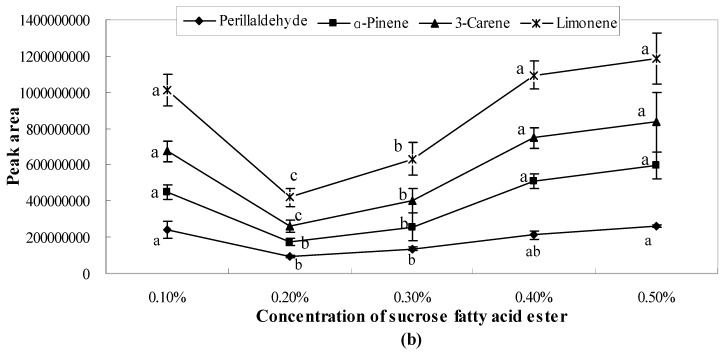
Effects of sucrose fatty acid ester on the release of ethyl acetate, (*E*)-2-hexenal, carvone (**a**) and perillaldehyde, α-pinene, 3-carene, limonene (**b**). Significant differences (*p* < 0.05) between different concentrations are performed by letters on the same polylines. Where the letters on the same polyline are the same, there is no significant difference between different concentrations.

**Figure 4:**
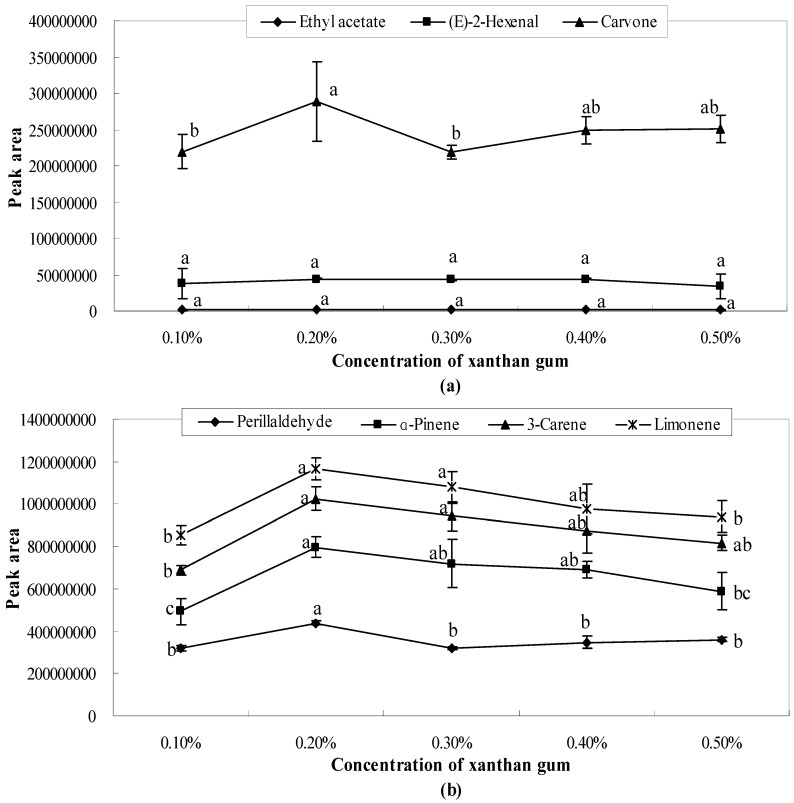
Effects of xanthan gum on the release of ethyl acetate, (*E*)-2-hexenal, carvone (**a**) and perillaldehyde, α-pinene, 3-carene, limonene (**b**). Significant differences (*p* < 0.05) between different concentrations are performed by letters on the same polylines. Where the letters on the same polyline are the same, there is no significant difference between different concentrations.

The present study found that there existed bonding interactions between emulsifier molecules and aroma molecules, which thereby reduce the concentration of free aroma molecules in aqueous/oil phase and restrict the transfer of aroma molecules from the oil to water phases thus, retaining the aroma. The results showed that the peak areas of ethyl acetate and (*E*)-2-hexenal were one or two orders of magnitude smaller than those of the other studied aroma compounds (3-carene, α-pinene, limonene, carvone, perillaldehyde). As shown in Figure 1, Figure 2 and Figure 3, the release of ethyl acetate was significantly (*p* < 0.05) affected by the concentration of emulsifiers, while no significant differences were observed in different concentrations of xanthan gum solutions (Figure 4). Additionally, the release amount of this compound was two or three order of magnitude lower than those of other aroma compounds. This result indicates that compared with other aroma compounds (3-carene, α-pinene, limonene, carvone, perillaldehyde), ethyl acetate was more likely to be conserved in the solution system (including the thickening agent, emulsifying agent, or a combination of both). Among all seven aroma compounds, the amount of limonene released was the highest. The lowest release amount of this compound was observed in the presence of 0.4% soybean lecithin. The delayed aroma release was mainly caused by the binding of flavours to emulsifier molecules.

The concentration gradient (0.1%, 0.2%, 0.3%, 0.4%, and 0.5%) of Tween 80 affected the release of aroma compounds. As shown in Figure 1a, the release of (*E*)-2-hexenal and carvone showed significant changes for different concentrations of Tween 80 solutions. The amount of ethyl acetate was relatively small, and the peak area of the released ethyl acetate reached the highest level in 0.3% Tween 80 solution. The other three aroma compounds (3-carene, α-pinene, limonene) also showed the highest peak areas in 0.3% Tween 80 solution (Figure 1b), while all seven aroma compounds showed the lowest peak areas when the concentration of Tween 80 was 0.1%.

The concentration gradients (0.1%, 0.2%, 0.3%, 0.4%, and 0.5%) of soybean lecithin and sucrose fatty acid ester obviously affected the release of these seven aroma compounds. ANOVA revealed a significant effect of different concentrations on aroma release (Figure 2). When the concentration of soybean lecithin was 0.1%, 0.3%, and 0.4%, the peak areas of each aroma compound were much closer to each other. However, when the concentration of soybean lecithin was 0.2% and 0.5%, a peak value that was significantly higher than that of other peak areas was observed.

All the aroma compounds had the largest release capacity when the concentration of soybean lecithin was 0.2%. When the concentration was 0.4%, the peak areas of various aroma compounds were the lowest. This implied that when the concentration of soybean lecithin was 0.2%, the release of the aroma compounds was promoted, whereas when the concentration was 0.3% and 0.4%, the release of the aroma compounds was considerably inhibited (Figure 2).

The peak area of sucrose fatty acid ester showed a high fluctuation range, indicating that the concentration of sucrose fatty acid ester considerably influenced aroma release. When the concentrations of sucrose fatty acid ester were 0.1%, 0.4%, and 0.5%, the peak area values of each aroma compound had no significant differences (*p* > 0.05). However, the peak area values were considerably low at the concentration of 0.2% and 0.3%. This indicated that the release effect was inhibited. Moreover, when the concentration of sucrose fatty acid ester was 0.2%, all of the aroma compounds showed the lowest release amount (Figure 3).

### 2.2. Influence of Xanthan Gum on Aroma Release

As shown in Figure 4, there were no significant differences (*p* > 0.05) in the peak areas of ethyl acetate and (*E*)-2-hexenal at different concentrations of xanthan gum solutions. This indicated that the concentration of xanthan gum had little influence on these two aroma compounds. As shown in Figure 4a, when the concentration of xanthan gum solution is 0.1% and 0.3%, the quantities of carvone and perillaldehyde released are the lowest; whereas when the concentration was 0.2%, the quantities released were the highest. However the release of ethyl acetate and (*E*)-2-hexenal had the lowest peak areas in 0.5% xanthan gum solution. In Figure 4b, the peak area was the lowest in 0.1% xanthan gum solution, which is approximately 0.5%. After 0.2%, the quantity of α-pinene, 3-carene, and limonene released exhibited a decreasing trend.

The effect of xanthan gum on aroma release was attributed to the variation of xanthan gum concentration at the oil-water interface, which resisted the transfer of most flavours to the headspace. This inhibition effect of xanthan gum resulted from its more distinctive hydrophobic character compared with other hydrocolloids; thus, the hydrogen bonding between xanthan gum and hydrophilic compounds would influence aroma release [18,21]. This observation was consistent with the fact that xanthan gum is an ionic polysaccharide comprising a β-(1,4)-d-glucopyranosyl backbone with ionized trisaccharide side chains comprising two mannose residues and one glucuronic acid residue. Xanthan gum is a multifunctional biological polymer with high molecular weight and a good deal of free carboxyl groups. Because of these active sites, xanthan gum exhibits great water absorption and water-holding capacity. When dispersed in aqueous solutions, xanthan gum transforms from an ordered double helix to a complex polymer through the action of hydrogen bonds and polymer entanglement. Xanthan gum molecules form a tenuous three-dimensional network. Bylaite *et al*. [18] found that the ordered conformation of xanthan gum gives a structure homologous to a double helix, which may create a “hydrophobic cavity” and can entrap aroma compounds. This ordered conformation plays an important role in solution viscosity [22]. Increasing the xanthan gum concentration enhanced the viscosity and thus retarded the release of aroma compounds. Therefore, the minimal variation of xanthan gum content was able to induce aroma release because of the complex composition and structure of xanthan gum [18,21,23].

### 2.3. Influence of Different Emulsifier Systems with Added Thickener on Aroma Release

Analysis of variance showed that there was no significant difference on the release of ethyl acetate between different concentrations of composite systems. However there were significant differences with the other aroma compounds. By analysing the changes of peak areas in mixed systems, it was observed that the combination of sucrose fatty acid ester and xanthan gum showed the maximum reduction. This finding indicated that the mixture of sucrose fatty acid ester and xanthan gum can effectively reduce the release of aromas. The interaction between sucrose fatty acid ester and xanthan gum may have promoted the formation of inclusion compounds and thus reduced the amount of aroma released. 

**Figure 5:**
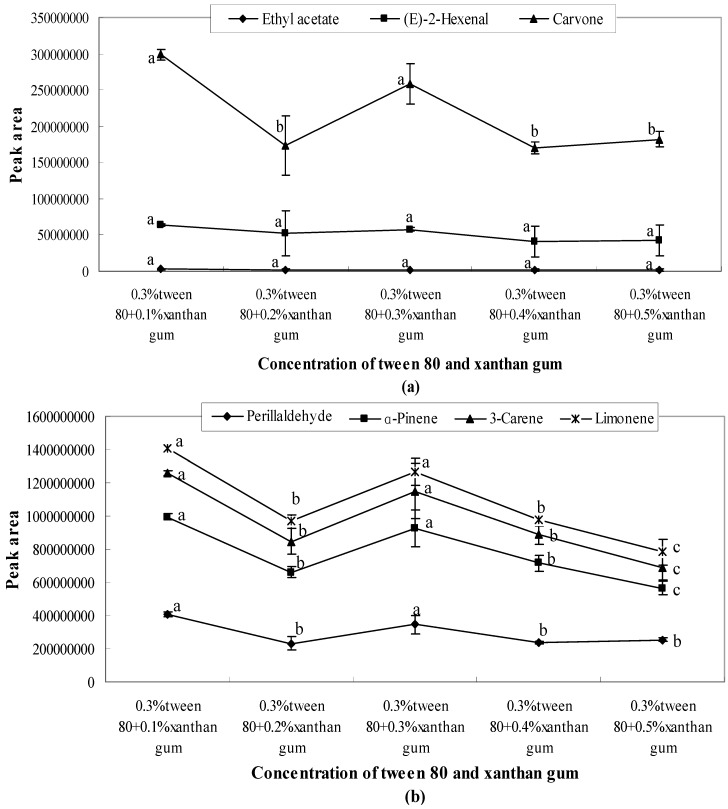
Effects of Tween 80 and xanthan gum on the release of ethyl acetate, (*E*)-2-hexenal, carvone (**a**) and perillaldehyde, α-pinene, 3-carene, limonene (**b**). Significant differences (*p* < 0.05) between different concentrations are performed by letters on the same polylines. Where the letters on the same polyline are the same, there is no significant difference between different concentrations.

**Figure 6:**
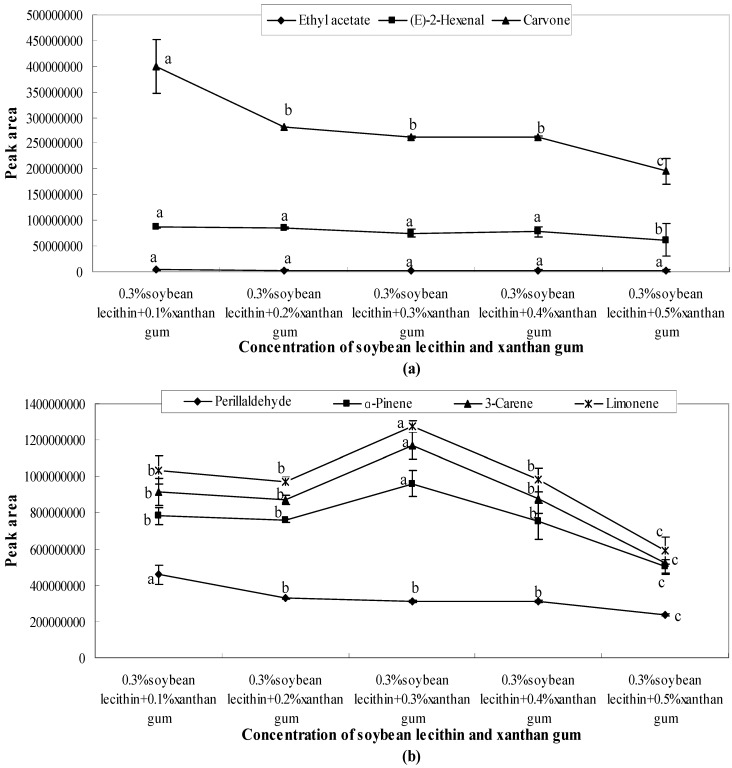
Effects of soybean lecithin and xanthan gum on the release of ethyl acetate, (*E*)-2-hexenal, carvone (**a**) and perillaldehyde, α-pinene, 3-carene, limonene (**b**). Significant differences (*p* < 0.05) between different concentrations are performed by letters on the same polylines. Where the letters on the same polyline are the same, there is no significant difference between different concentrations.

**Figure 7:**
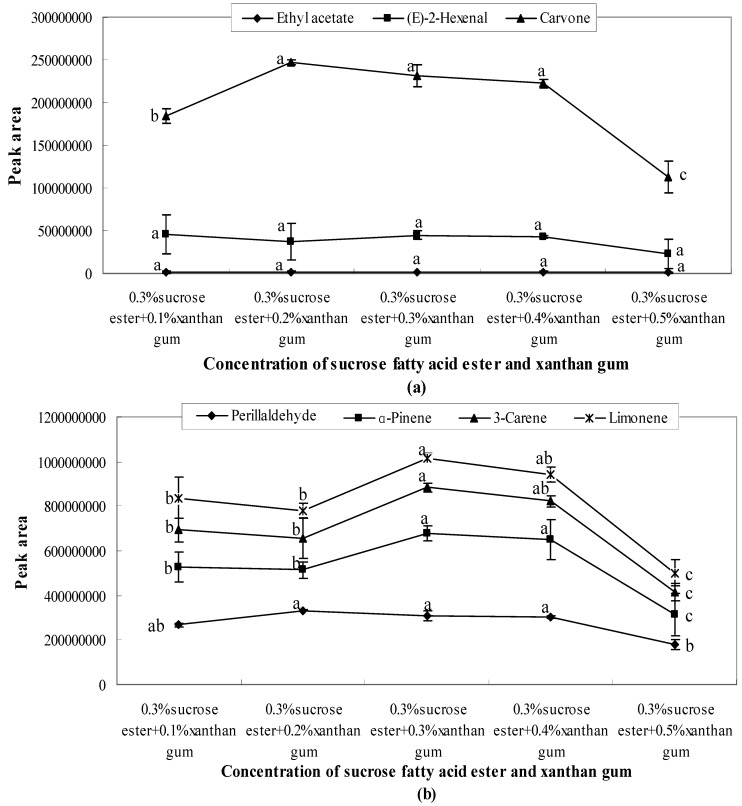
Effects of sucrose fatty acid ester and xanthan gum on the release of ethyl acetate, (*E*)-2-hexenal, carvone, perillaldehyde (**a**) and α-pinene, 3-carene, limonene (**b**). Significant differences (*p* < 0.05) between different concentrations are performed by letters on the same polylines. Where the letters on the same polyline are the same, there is no significant difference between different concentrations.

As shown in Figure 5, Figure 6 and Figure 7, the combination of emulsifiers and xanthan gum increased the quantity of aroma released compared with single emulsifier systems. However, as the amount of supplemented xanthan gum increased, the amount released showed a downward trend. Meanwhile, a lower amount was released at a higher concentration of xanthan gum. The influence of different emulsifier systems with xanthan gum on aroma release was analysed, and a comparison and analysis of the peak areas obtained from various aroma compounds and solution systems were carried out.

The following conclusions could be drawn from the trend charts:

(1) When 0.3% Tween 80 was mixed with different concentrations of xanthan gum (0.1%, 0.2%, 0.3%, 0.4%, and 0.5%), the quantity released with 0.2% xanthan gum was lower than that of other concentrations as shown in Figure 5. After 0.3%, the amount released showed a downward trend.
(2) The quantity of carvone released presented a significant downward trend, seen in Figure 6a. With 0.3% soybean lecithin, the quantities of carvone, perillaldehyde, and (*E*)-2-hexenal released were restrained with the increase of xanthan gum concentration. All these compounds had the lowest peak area when the concentration of xanthan gum was 0.5%. However, the amount of ethyl acetate released was not significantly changed. As shown in Figure 6b, the amount released presented a significant (*p* < 0.05) downward trend after 0.3%.
(3) When 0.3% sucrose fatty acid ester was mixed with xanthan gum at different concentrations (0.1%, 0.2%, 0.3%, 0.4%, and 0.5%), aroma release showed a sharp decline after 0.4% (Figure 7). The lowest peak areas were observed when the concentration of xanthan gum was 0.5%. After 0.4%, the interactions between emulsifiers and xanthan gum influence the aroma release by entrapping aroma molecules or generating hydrogen bonds with aroma molecules [24].

Analysis of variance showed that concentration had a significant effect (*p* < 0.05) on aroma release, indicating that binding between these aroma compounds and emulsifiers was significant. Xanthan gum and emulsifiers considerably influenced aroma retention and release. Variations in aroma release were found because of the different concentrations of xanthan and emulsifiers. Different emulsifiers and complexes showed different surface properties that tend to interact with different aroma molecules. Furthermore, the different characteristics of emulsifiers such as molecular weight, viscosity, droplet size, emulsion type can make a difference on aroma release to different degrees [25].

Overall, emulsifiers considerably affected the behavior of aroma release, due to the composition and structure of emulsifiers, specific interactions such as hydrogen bonding, and the formation of an inclusion compound between emulsions and aroma molecules [5,26]. The binding effect reduced the concentration of free aroma in the aqueous/oil phase and limited the transmission of aroma molecules from the oil to the water phase; this reduced the transmission rate at the emulsion–gas interface [27,28].

## 3. Materials and Methods 

### 3.1. Reagents and Reference Samples

Three different emulsifiers were used in this study, namely, Tween 80, soybean lecithin, and SE-15 sucrose fatty acid ester. Tween 80 was supplied by Sinopharm Chemical Reagent Co., Ltd. (Shanghai, China), soybean lecithin was supplied by Biosharp Company (Hefei, China), and SE-15 sucrose fatty acid ester was supplied by Hangzhou Rui Lin Chemical Co. Ltd. (Hangzhou, China). Seven different aroma compounds were used in this study, namely, 3-carene, ethyl acetate, perillaldehyde, (*E*)-2-hexenal, carvone, α-pinene (Sigma Aldrich, Saint Louis, MO, USA), and limonene (Tokyo Chemical Industry, Tokyo, Japan). Xanthan gum (Shanghai Jingchun Biochemical Technology Co., Ltd., Shanghai, China) was used to prepare different emulsifier systems to which the thickener was added. Anhydrous ethanol (Sinopharm Chemical Reagent Co., Ltd., Shanghai, China) was added to dissolve the aroma compounds. 

### 3.2. Preparation of the Mother Liquor of the Aroma Substance

The selected aroma compounds were dissolved using anhydrous ethanol as the solvent and were quantified by 25 mL volumetric flask. Aroma components were added using a 100 µL disposable pipette. To avoid the contamination of reagents, the used pipet tip was constantly changed, and disposable plastic droppers and plastic sealing membranes were used. After preparing the mother liquor, the volumetric flask was covered by a bottle stopper and its neck was sealed with a 1 cm-wide plastic sealing membrane to prevent the aroma compounds from volatilizing in normal temperature and causing experimental errors. 

### 3.3. Preparation of Single Emulsifier System

Xanthan gum, Tween 80, sucrose fatty acid ester, and soybean lecithin were prepared into different solutions with concentration gradients of 0.1%, 0.2%, 0.3%, 0.4%, and 0.5%. The emulsifiers were accurately weighed and dissolved in distilled water. During dissolution, a benchtop closed electric furnace (Hengrui Keyi Instrument Co. Ltd., Tianjin, China) was used to heat the solution, which was constantly stirred using a glass rod. The solution was cooled to room temperature after complete dissolution. The volume was kept constant to achieve a final volume of 100 mL.

### 3.4. Preparation of Different Emulsifier Systems Supplemented with Thickener

Sucrose fatty acid ester, soybean lecithin, and Tween 80 were prepared as 0.3% solutions, to which 0.1%, 0.2%, 0.3%, 0.4%, and 0.5% xanthan gum were added. Emulsifiers and xanthan gum were accurately weighed, dissolved in distilled water, and heated using the benchtop closed electric furnace. Sucrose fatty acid ester, soybean lecithin, or Tween 80 was first dissolved, and xanthan gum with different concentration gradients was added. The solution was cooled to room temperature after complete dissolution. The volume was kept constant to achieve a final volume of 100 mL.

### 3.5. Extraction and Analysis of Aroma Substances

A solid-phase microextraction (SPME) manual device equipped with 50/30 µm divinylbenzene/carboxen/polydimethylsiloxane (DVB/CAR/PDMS) fiber (Supelco, Bellfonte, PA, USA) was used to extract the aroma compounds. The fiber was conditioned in a GC injector port at 270 °C for 1 h before use. Afterward, 10 mL of the samples was placed in a 25 mL vial containing a microstirring bar. The samples were equilibrated at 40 °C for 15 min and extracted using the DVB/CAR/PDMS fiber for 40 min at the same temperature with continuous stirring. After volatiles were extracted, the fiber was inserted into the GC injection port to desorb the analytes for 5 min [29].

### 3.6. GC-MS Analysis

Volatile compounds were subjected to GC analysis on an Agilent 6890N GC coupled to an Agilent 5975B mass spectrometer (Agilent Technologies, Santa Clara, CA, USA) and equipped with a J & W HP-5MS fused silica capillary column (30 m × 0.25 mm i.d., 0.25 μm film thickness). Mass spectral ionization was set at 230 °C. The mass spectrometer was operated in an electron ionization mode at a voltage of 70 eV. The flow rate of helium on the HP-5MS column was 1.2 mL/min. Analysis was conducted in a splitless mode. Injector temperature was 250 °C. The column was initially maintained at 40 °C for 3 min; temperature was then increased from 40 °C to 160 °C at 3 °C/min, maintained at 160 °C for 2 min, and finally increased to 220 °C at a rate of 8 °C/min. Temperature was maintained at 220 °C for 3 min.

### 3.7. Statistical Analysis

Significant differences between the various concentrations on aromas release was performed by one-way ANOVA in SPSS 19.0 for Windows (SPSS Inc., Chicago, IL, USA). The significance level was *p* < 0.05 throughout the study. 

## 4. Conclusions

This study demonstrated the effect of different emulsifiers and emulsifiers with xanthan gum on aroma release. Indicators showed that the concentration of emulsifiers affected aroma release, but that the concentration of xanthan gum should be considered as well. The present study showed that the emulsifiers and xanthan gum considerably affected aroma release because the properties of emulsion were greatly determined by the emulsifiers. Moreover, there exist bonding interactions between emulsifier molecules and aroma molecules, thus restricting the transfer of aroma molecules. As the experimental results showed, sucrose fatty acid ester, soybean lecithin, Tween 80, and xanthan gum influenced the release of aroma compounds to varying extents. Moreover, the release amount of aroma compounds was different when their concentration was different. When xanthan gum was added into the emulsifier system, the amount of aroma released was increased. When the concentration of xanthan gum was increased, aroma release presented a downward trend. These results demonstrated that the interaction between the emulsifier and xanthan gum changed the release amount of aroma compounds. Further studies should be conducted to explore the more complicated interactions between aroma compounds and emulsifiers.
